# Widespread introgression of mountain hare genes into Fennoscandian brown hare populations

**DOI:** 10.1371/journal.pone.0191790

**Published:** 2018-01-25

**Authors:** Riikka Levänen, Carl-Gustaf Thulin, Göran Spong, Jaakko L. O. Pohjoismäki

**Affiliations:** 1 Department of Environmental and Biological Sciences, University of Eastern Finland, Joensuu, Finland; 2 Molecular Ecology Group, Department of Wildlife, Fish, and Environmental Studies, Swedish University of Agricultural Sciences, Umeå, Sweden; 3 Department of Anatomy, Physiology and Biochemistry, Swedish University of Agricultural Sciences, Uppsala, Sweden; 4 Forestry and Environmental Resources, College of Natural Resources, North Carolina State University, Raleigh, North Carolina, United States of America; Universita degli Studi di Roma La Sapienza, ITALY

## Abstract

In Fennoscandia, mountain hare (*Lepus timidus*) and brown hare (*Lepus europaeus*) hybridize and produce fertile offspring, resulting in gene flow across the species barrier. Analyses of maternally inherited mitochondrial DNA (mtDNA) show that introgression occur frequently, but unavailability of appropriate nuclear DNA markers has made it difficult to evaluate the scale- and significance for the species. The extent of introgression has become important as the brown hare is continuously expanding its range northward, at the apparent expense of the mountain hare, raising concerns about possible competition. We report here, based on analysis of 6833 SNP markers, that the introgression is highly asymmetrical in the direction of gene flow from mountain hare to brown hare, and that the levels of nuclear gene introgression are independent of mtDNA introgression. While it is possible that brown hares obtain locally adapted alleles from the resident mountain hares, the low levels of mountain hare alleles among allopatric brown hares suggest that hybridization is driven by stochastic processes. Interspecific geneflow with the brown hare is unlikely to have major impacts on mountain hare in Fennoscandia, but direct competition may.

## Introduction

Species boundaries are frequently challenged by lineage divergence and hybridization [1]. Diverged lineages (i.e. species) are maintained by barriers to gene flow that vary in strength over time, space, or the genome [2]. For closely related species, the barrier may be permeable [3–5]. Changes in ecology, behavior, population dynamics and distribution may all result in increased levels of spatial and temporal sympatry between closely related species, leading to an increased frequency of hybridization events. These often have profound effects on a wide range of individual- and population level processes. At the individual level, hybridization may affect fitness by creating novel combinations of traits adapted to different environments [6–8]. At the population and species level, hybridization may lead to the introgression of new genetic variation, affecting the diversity within and between species [9].

As the genetic variation provided by hybridization is exposed to selection, hybrids experience an increased, decreased or neutral selection effect, and the effect of this selection may vary in direction and strength across the genome and context [1,2,10]. The signs of introgression are therefore unevenly distributed throughout the genome, with some regions showing selection for, and other against, the introduced genetic material [11,12]. At the population level, introgression typically occurs in hybridization zones at species boundaries, resulting in genetic gradients across the population. At the species level, patterns of introgression are also often highly variable and asymmetric, and may have a strong directional bias towards one of the species in the pair [13]. The outcomes and signatures of hybridization are thus highly contingent on temporal and spatial scales, phylogenetic and demographic relationships and the particulars of the hybridization events. For example, introgression could in theory erase a species boundary, thereby redistributing genetic variation, but without affecting the total amount of standing genetic variation. However, introgression might also accelerate speciation or colonization processes by providing adaptive alleles [14].

From a conservation perspective, introgression create challenges for management efforts, typically by blurring the species barrier [15], affecting the distribution and amount genetic variation, and obfuscating the delineation of appropriate management units [16,17]. Moreover, hybridization can lead to genetic swamping, where hybrids overwhelm or outcompete the rarer species, or to demographic swamping where inferior hybrids lead to a lower population growth rate [18]. In both cases, the loss of species or locally adapted populations is at risk. A contrasting view is that hybridization offers genetic rescue to genetically impoverished populations in risk of genetic meltdown or as a means for species to more rapidly adapt to ecological change [12,14]. Whether viewed as a threat or opportunity, hybridization clearly present challenges for conservation. For example how prevalent it is, which genetic and demographic effects it has, and whether can it be considered independent of or caused by anthropogenic factors. Improved genetic methods allow for more comprehensive understanding of the genomics of introgression and its effect on all levels of biological diversity.

In Europe, northern Fennoscandia represents the northernmost contact zone between brown hare (*Lepus europaeus* Pallas) and mountain hare (*Lepus timidus* L.). Benefitted by translocations and, potentially, climate change, the brown hare has been extending its distribution northwards since the 19^th^ century. In contrast, mountain hare populations have been in decline in Finland and southern Sweden [19,20]. Currently, brown hares are abundant in southern and central Sweden and Finland where they mainly exist in sympatry with the mountain hare. Previous research have shown transmission of mitochondrial DNA (mtDNA) from mountain hares into brown hare populations [21,22], where the genetic material is maintained for generations even in geographical areas where mountain hares have become extinct [21,23,24]. Because of the preservation of mtDNA linages the question has been raised whether this introgression has adaptive value or maintained simply due to stochastic demographic processes [13,21,25,26]. Increased frequency of introgressed mtDNA could happen through genetic drift in the extremity of the species distribution, where population density is low and population growth is rapid [27]. Once the population increases and expands its range, for example due to climate change, the introgressed mtDNA could also propagate and imprint into the local resident population, in particular if certain mtDNA lineages generate adaptive advantages. This is suggested to have happened in the Iberian peninsula, where mountain hare mtDNA has been introduced to brown hare population through repeated introgression along the expansion front of the brown hare after last glacial maximum [28]. Recent work done on Iberian hares, involving nuclear DNA markers, suggests that mtDNA introgression among *Lepus* is driven by demographic processes [29], facilitated by continuous changes of species distribution in the wake of quaternary climate oscillations [30], which could be generalized to explain the common nature of the phenomenon.

The overlap in distribution of mountain hares and brown hares in Fennoscandia and their concurrent population expansion and contraction, presents an outstanding opportunity to study ongoing genetic interactions between two closely related species adapted to different ecological conditions that are now changing rapidly.

Here we analyze 6833 SNP markers across the genome to quantify introgression among Finnish- and Swedish mountain hares and brown hares. We show introgression of mountain hare markers into brown hare, but not *vice versa*, to be frequent in areas of sympatry. The introgression was independent on levels of mtDNA introgression, confirming the highly asymmetric nature of the genetic interaction between the two species. Genotyping also revealed differences in the genetic diversity of brown hares from the two countries, as expected from their different colonization histories. Besides identifying new genetic markers for *Lepus*, our study provides evidence of genetic swamping by an invasive species (brown hare) at the expense of a resident species (mountain hare). As ecological change forces species to adapt by changes in distribution or behavior, the demographic and genetic consequences of increased levels of sympatry and hybridization are important to consider for management.

## Materials and methods

### Tissue samples and DNA isolation

**Fig 1:**
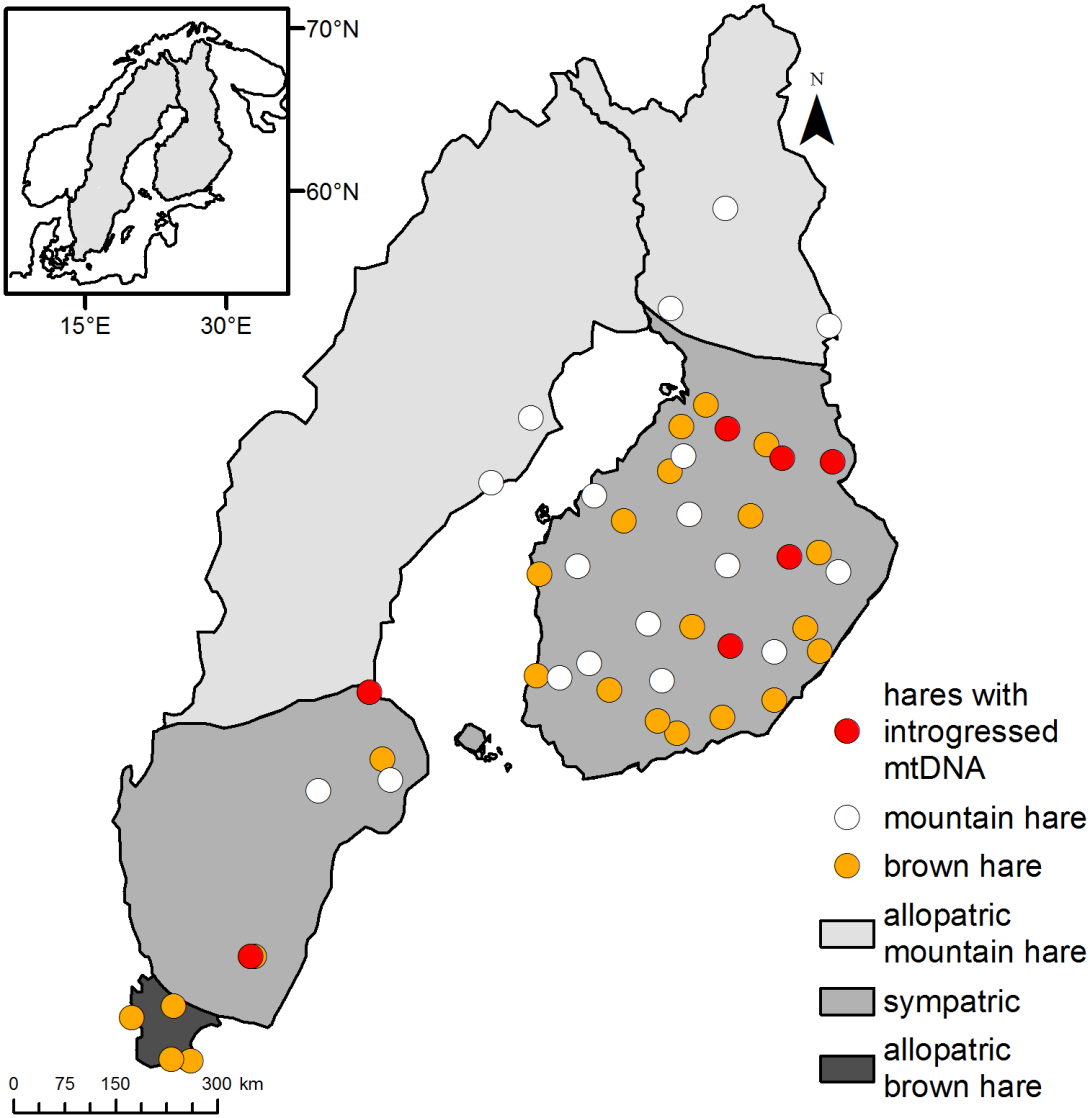
Sample distribution across Sweden and Finland.

For the genome-wide SNP analysis, DNA was isolated from 22 mountain hares and 27 brown hares (muscle from the base of the ear), including specimens with introgressed mtDNA and representing allopatric as well as sympatric populations almost throughout the full range of the species’ distribution in Sweden and Finland (Table 1, Fig 1). Hunters collected the specimens during normal hunting season, following the regional hunting seasons and legislation, using 12 to 20 gauge shotguns with 3–4 mm shot size. No animal was killed for research purposes only. All specimens were initially identified using morphological characters. The samples included in this study belong to a larger DNA collection of 904 Finnish and 1270 Swedish hares, sorted according to the species, country of origin, mtDNA haplotype as well as allopatric/sympatric occurrence. Populations at the extreme ends of the range, with high certainty of no contemporary contact with the other species, were assigned as allopatric populations (Fig 1). While specimens with conspecific mtDNA haplotype were selected randomly, specimens with introgressed mtDNA were intentionally included in to the study to test the degree of nuclear DNA admixture as well as to monitor for the accuracy of morphological species determination. As pointed out earlier, mtDNA introgression in hares is highly asymmetric, from mountain hare to brown hare and the transfer of mtDNA from brown hares to mountain hares have been considered as rare events and generally of less ecological or genetic significance [31]. DNA was isolated using Chelex^®^ 100 (Bio-Rad) method [32], following manufacturers’ recommendations.

**Table 1:** DNA samples used in the study by country, population and mtDNA genotype.

Species	Country	Population	mtDNA	*N*
*L*. *timidus*	FIN	Allopatric	Conspecific	3
*L*. *timidus*	FIN	Sympatric	Conspecific	11
*L*. *timidus*	FIN	Sympatric	*L*. *europaeus*	3
*L*. *timidus*	SWE	Allopatric	Conspecific	2
*L*. *timidus*	SWE	Sympatric	Conspecific	2
*L*. *timidus* (Hybrid)	SWE	Sympatric	*L*. *europaeus*	1
*L*. *europaeus*	FIN	Sympatric	Conspecific	18
*L*. *europaeus*	FIN	Sympatric	*L*. *timidus*	2
*L*. *europaeus*	SWE	Allopatric	Conspecific	4
*L*. *europaeus*	SWE	Sympatric	Conspecific	2
*L*. *europaeus*	SWE	Sympatric	*L*. *timidus*	1

Note that the *L*. *timidus* with *L*. *europaeus* mtDNA from Sweden was revealed to be a first generation hybrid by SNP genotyping. FIN: Finland, SWE: Sweden.

### mtDNA genotyping

Mitochondrial DNA was genotyped from all samples by amplifying a 669 bp cytochrome b (*Cytb*) fragment using polymerase chain reaction (PCR) and LCYTBF and LCYTBR primers [33]. The identity of PCR for the species-specific reference products was confirmed by Sanger sequencing (GATC Biotech AG, Germany). The PCR products were digested with *Alu*I restriction enzyme and run over a 3% high resolution agarose gel electrophoresis to reveal restriction fragment lengths and the subsequent genotype [23,33].

### SNP genotyping and analysis

199,693 SNPs were analyzed using the commercially available GeneChip^™^ Rabbit Gene 1.0 ST Array genotyping DNA-chip (Thermo Fischer Scientific #902238). Analyses of population structure and -admixture were performed using 6833 polymorphic loci informative for both hare species (S1 File). The genotype data together with the detailed specimen information can be accessed at the Dryad Data repository (doi:10.5061/dryad.n70q6). Basic population genetic analyses, such as computing allele frequencies and testing for Hardy-Weinberg equilibrium, were performed using Arlequin 3.5. [34]. The PCA analyses to cluster specimens based on the SNP genotypes, was performed in R using the package SNPRelate found at https://bioconductor.org/biocLite.R. Genetic diversity in the hare species was assessed using STRUCTURE 2.3.4 [35,36], using the admixture model, 2–10 populations (*K*), three iterations with 1 million MCMC repetitions for burnin period of 500,000 [29]. Sampling locations were used as prior information (LOC prior) to expose any shallow population structures. Iterations were matched using CLUMPP v1.1.2 [37]. *K* or the best number of populations was obtained using STRUCTURE HARVESTER [38]. The detected 192 species-specific SNPs (see Results and Discussion) were used to estimate the degree and direction of hybridization using NEWHYBRIDS software [39], implementing a Gibbs sampler to estimate the posterior probability for individuals falling into defined hybrid categories. The default Prior and Theta settings were used with 100,000 sweeps before and after burnin. Mitochondrial DNA haplotype was also included in the NEWHYBRID analysis as a haploid (“dominant”) marker.

## Results and discussion

The genotyping data enabled us to detect highly asymmetrical introgression of nuclear genes from *L*. *timidus* to *L*. *europaeus* at average level of 2% in regions of sympatry but being almost absent in allopatric populations. In total, 62,730 SNP genotypes had 100% call rate for the 49 samples and 6833 of these were polymorphic (Table 2, S1 and S2 Files). Although no phylogenetic dating of *Lepus* and *Oryctolagus* divergence has been made, comparative evidence from Ig gene sequences suggests that the two genera could be as distant as mice and rats [40], which split almost 12 Ma ago [41]. Although SNPs that have remained variable between the species during long evolutionary histories, could be enriched in loci under balancing selection, only 277 loci in brown hare and 109 in mountain hare were not in Hardy-Weinberg equilibrium (S3 File). It may be that the number of analyzed loci has been effective in picking up rare neutral loci that have retained variation across genera. It should be noted that the shared polymorphisms are rare, resulting in low minor allele frequencies in the two species (Table 2). Interestingly, none of the alleles was monomorphic in one species, but typically present at low frequency also in the other one. This might not be surprising, as drift alone would require 9–12 *N*_e_ (= size of the effective population) generations to make the diverging species monophyletic at more than 95% of the loci during allopatric speciation [42]. We identified only 192 SNPs having allele frequencies of over 80% in one species but not in the other, which could be useful in species identification (S2 File).

**Table 2:** SNP heterozygosity levels and minor allele frequencies for the 6833 polymorphic SNPs by hare species and country of origin.

Species	Country	*N*	*Hz* loci	MAF	Mean *Hz*
*L*. *timidus*	FIN	16	0.39	0.05	0.08
*L*. *timidus*	SWE	5	0.26	0.05	0.09
*L*. *europaeus*	FIN	20	0.45	0.07	0.11
*L*. *europaeus*	SWE	7	0.55	0.09	0.16

FIN: Finland, SWE: Sweden, *Hz* loci: frequency of heterozygous loci, MAF: Minor Allele Frequency, *Hz*: Heterozygosity.

### Genetic diversity in Fennoscandian hare species

**Fig 2:**
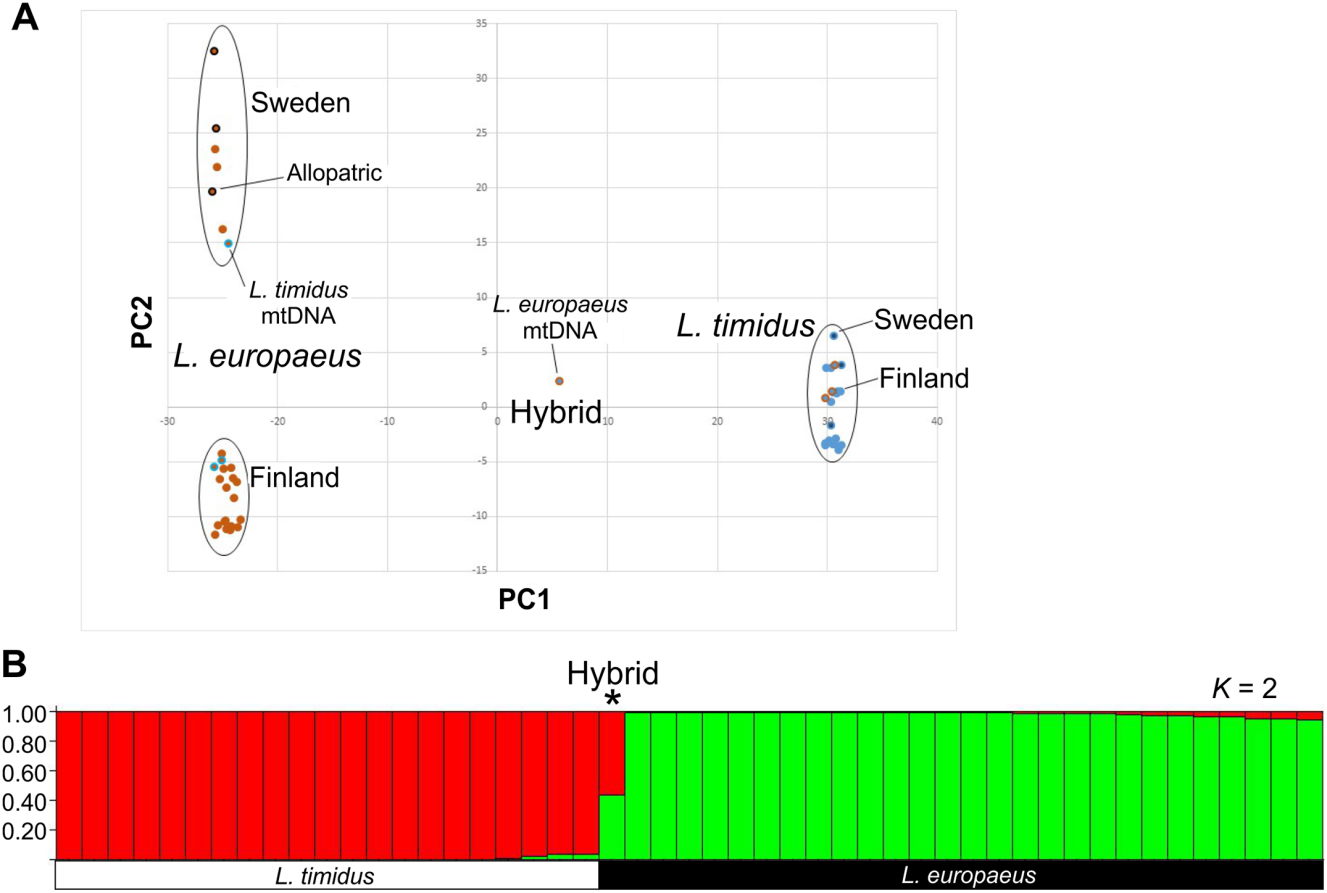
Genetic similarities and population structures among Fennoscandian hares. (A) Clustering of the 48 genotyped specimens using principal component analysis (PCA) of the SNP data. While the Swedish and Finnish brown hare populations are genetically distinct, the mountain hares from the two countries clearly belong to the same Fennoscandian population. Orange fill: Brown hare, Light blue fill: Finnish mountain hare specimens, Dark blue fill: Swedish mountain hares. Color of the outer ring marks mtDNA genotype whereas allopatric brown hares are marked with black outer ring. Note how the individual hybrid specimen is with brown hare mtDNA is located midway of the two species. Specimens with introgressed mtDNA are otherwise embedded among conspecific samples. (B) Population structure among all 48 samples. A high degree of differentiation between the two species apart for the one hybrid (*). The yellow color, representing typical mountain hare allele combinations, trails into the brown hare clusters. Two ancestral populations (*K* = 2) was chosen to illustrate hybridization between the two species.

Although the small number of polymorphic loci, ambiguity of chromosomal locations and relatively low levels of minor alleles as well as mean heterozygosity, resulting from using across species SNP array, disallowe more detailed population genetic analyses, the 6833 loci do pick up differences between the two hare species and work as a proxy to compare genetic diversity in Sweden and Finland. Interestingly, the Swedish brown hares show higher heterozygosity levels and minor allele frequencies than their Finnish counterparts, whereas the mountain hares from the two countries do not differ (Table 2). The high heterozygosity in Swedish brown hares correlate also with the genetic diversity as revealed by grouping the specimens by genetic similarity using PCA, where the Swedish brown hare genotypes are more scattered compared to the Finnish specimens or mountain hares (Fig 2A).

It is likely that the overall higher heterozygosity in the brown hares compared to mountain hares does not only result from the biased gene flow from mountain hares to brown hares. For example, the country-specific differences in the genetic diversity among brown hares can be explained by the differential colonization histories in the two countries. Brown hares were first introduced to Sweden in 1858 to the island of Ven in the Öresund straight between Sweden and Denmark [43]. After translocations to the Swedish mainland, the species was well established in the south in the late 19^th^ century and subsequently expanded to southern and central Sweden [19]. The brown hare is still expanding northwards in Sweden [44].

Swedish brown hares show high mtDNA haplotype diversity, likely due to admixture due to repeated introductions of brown hares from several geographical areas of the native (continental) range of brown hares [45]. This high mtDNA diversity has likely been accompanied by a diversity of nuclear DNA, explaining the differences in heterozygosity (Table 2), as well as in genetic distance (Fig 2A) between Swedish and Finnish brown hares. In addition, the continuous introgression of mountain hare mtDNA to brown hares in Sweden [21] has led to transfer of nuclear DNA over the species barrier [46], further adding to the genetic composition of brown hares in Sweden. In contrast, the brown hare has established in Finland through spontaneous immigration, mainly during the 20^th^ century in association with a general, northeastward expansion of brown hares noted by Thenius [47]. In Finland, and elsewhere, this expansion has likely been assisted by translocation and supplementary releases, although this has not been properly documented.

Thus, the genotypic differences in SNPs observed between Swedish and Finnish brown hares in the current study, may reflect the differences in the population history of the recurrent populations. The composition of the Swedish brown hare gene pool is a result of admixture of hares from different areas in continental Europe [39] along with introgression from mountain hares [21,51], while Finnish brown hares likely reflect the stepwise and/or gradual expansion pattern observed at the edge of a species distribution [31,48], in association with introgression from mountain hares [49].

### Admixture between mountain hare and brown hare

Population structure analysis enabled us to identify species-specific genetic markers and confirm introgression between mountain hares and brown hares. Based on the NEWHYBRID analysis of the species-specific markers, introgression was highly asymmetric, with brown hares being classed as backcrosses whereas all mountain hares included in the study were ranked as purebred (S4 File). The average degree of backcrossing to *L*. *europaeus* background cannot be reliably estimated as retention of ancestral polymorphism because of incomplete lineage sorting (ILS) and secondary gene flow through introgression, produce very similar patterns of shared genetic diversity between two species [50,51]. For example, after five generations of backcrosses, only 1.6% of introgressed alleles would be retained, low enough number to be sampled by chance in case of 192 markers. Interestingly, individuals with introgressed mtDNA did not differ significantly from the other sympatric specimens. This is likely to reflect a situation, where hybridization in the areas of sympatry is frequent, but that the most of the mountain hare genotypes are not selectively maintained in the brown hare population but are diluted out in the regions were mountain hares are not present [21,26,28,29,33,52].

As a curiosity, the mountain hare like hybrid (Table 1 and S4 File) having *L*. *europaeus* mtDNA represented 0.56 *timidus* and 0.44 *europaeus* share of ancestral populations in STRUCTURE analysis (Fig 2). Although it had only 12% total marker heterozygosity, representing a midway between the mean heterozygosities among the species (Table 2). 86% of the 192 species-specific loci (S2 File) were heterozygous (S5 File). When also the NEWHYBRID analysis gave posterior probability value of 1.00 for F1 hybrid assignment in (S4 File)., we suggest that this individual is a genuine first generation hybrid between a female brown hare (mtDNA donor) and male mountain hare. We were also able to confirm that the few other examples of brown hare mtDNA being introgressed into mountain hare were genuine observations and not misidentifications by hunters (Fig 2, S4 File). Interestingly, these specimens had only traces of brown hare specific nuclear markers in contrast to the patterns of genetic imprint among brown hares with mountain hare mtDNA (S4 File).

## Concluding remarks

Our study represents the first attempt to assess nuclear gene introgression in hares using a SNP-chip designed for a related species (the rabbit) and, more specifically, it is the first such study focused on mountain hares and brown hares in Fennoscandia. While it is clear that the next generation methods, such as Double Digest Restriction Associated DNA (ddRAD) sequencing [53], provide more powerful alternatives for genotyping species without prior knowledge of their genomes, exploitation of existing and standardized genome-wide genotyping methods can sometimes be cost-efficient and fast, providing useful data for population genetic analysis. The low levels of minor allele frequencies and heterozygosity among the 6833 polymorphic markers indicate that the detected SNPs are not optimal for detailed population analysis. For example, a commercial dog SNP panels typically give 0.30–0.40 heterozygosity rates within breed [54]. However, SNPs that show relatively little variation could constitute highly species-specific markers. Such conservative polymorphisms could be more useful in comparative studies than common polymorphisms used to differentiate individuals. Therefore, we believe that the discovered SNPs in this study prove to be useful as a future resource for hare population genetics.

Our results provide support for the preferential introgression of nuclear genes from *L*. *timidus* to *L*. *europaeus* in Fennoscandia, as previously reported for mtDNA [21,22,49]. Based on the mtDNA evidence, introgression is most frequent at the leading edge of brown hare range expansion [21,49]. Contrary to the comprehensive study using 100 SNPs obtained from RNA-seq transcriptome data from 314 *L*. *granatensis* specimens, assessing the patterns of the historical introgression events [29], our study aimed to validate the degree of nuclear marker admixture during ongoing contact between the two species in Fennoscandia. While it is likely that mtDNA introgression and preservation can be explained by demography [29], it is plausible that brown hare could also obtain locally adapted alleles from the resident mountain hares, which are expected to represent only a tiny fraction of the total genome. A limitation of transcriptome studies is that they provide expression data and transcript genotypes only from the sampled tissue, such as kidney or liver, and might not be able to capture genes influencing traits such as coat color variation [55,56], muscle metabolism [57], diet specialization [58] and immunity [59]. At present, comprehensive surveys of this type of adaptive variation in species with unknown genomes are generally not possible. However, already the current next generation sequencing methods enable the simultaneous genotyping of dozens of candidate gene loci, enabling the correlation of genotypes with phenotypes and the detection of single adaptive alleles. As the costs of whole genome sequencing are constantly dropping, it is likely that *ad hoc* comparisons of genomes become the standard practice in population genetics in the near future.

Although brown hare may outcompete mountain hare under certain conditions [19,60], the two species has coexisted in sympatry in most of Fennoscandia for decades. While hybridization between sympatric species can be a threat to endangered species, theoretically in less than five generations [61], genetic introgression in our hare-model system has significance mainly to the receiving brown hare population. Shortened snow-covered season, resulting in a dramatic mismatch with the protective white, may pose an additional threat to mountain hares regardless of brown hare’s range expansion. Although the mountain hare populations might be contracting at the southern edges of the species’ distribution [20], for a cold-adapted species, mountain hare has shown a remarkable resilience in the past [62] and is likely survive in Fennoscandia in the future.

## Supporting information

S1 File Probe sequences for the SNP markers used in the study. (XLSX)

S2 File Allele frequencies for all analyzed loci used and a list species diagnostic SNP markers. (XLSX)

S3 File List of loci not in Hardy-Weinberg equilibrium in brown hare and mountain hare. Species on separate spreadsheets.
(XLSX)

S4 File Posterior probabilities for different hybrid classes based on NEWHYBRID analysis. (XLSX)

S5 File F1 hybrid genotyping using the species-specific loci listed in S2 File. (XLSX)
